# Ovalbumin-induced plasma interleukin-4 levels are reduced in ceramide kinase-deficient DO11.10 RAG1^-/- ^mice

**DOI:** 10.1186/1476-511X-9-1

**Published:** 2010-01-06

**Authors:** Satoru Niwa, Nicole Urtz, Thomas Baumruker, Andreas Billich, Frédéric Bornancin

**Affiliations:** 1 Novartis Institutes for BioMedical Research, Brunnerstrasse 59, A-1235 Vienna, Austria; 2 Current address: German Heart Centre Munich, Lazarettstrasse 36, D-80636 München, Germany

## Abstract

Ceramide kinase (CERK) produces the bioactive lipid ceramide-1-phosphate (C1P) and is a key regulator of ceramide and dihydroceramide levels. It is likely that CERK and C1P play a role in inflammatory processes but the cells involved and the mechanisms used remain to be clarified. In particular, the impact of CERK on T-cell biology has not been studied so far. Here, we used *Cerk*^-/- ^mice backcrossed with DO11.10/RAG1^-/- ^mice to probe the effect of CERK ablation on T-cell activation. Levels of interleukin (IL)-2, IL-4, IL-5, IL-13, of tumor necrosis factor (TNF)-α, and of interferon (INF)-γ were recorded following ovalbumin challenge in vivo and using ovalbumin-treated splenocytes ex- vivo. Absence of CERK led to a significant decrease in the production of IL-4, thus suggesting that CERK may polarize T cells towards the T_H_2 cell subtype. However, the importance of CERK to T_H_2 cell biology will have to be investigated further because in a model of asthma, which is T_H_2-cell driven, *Cerk*^-/- ^mice responded like wild-type animals.

## Background

Ceramide kinase (CERK), together with sphingosine kinases (SPHK) 1 and 2, belongs to the diacyglycerol kinase family of lipid kinases. CERK is the only enzyme known to produce ceramide-1-phosphate (C1P) [1]. However, studies with CERK deficient (*Cerk*^-/-^) mice have shown that another route for production of C1P must exist, at least in mammals [2,3]. The best described signaling properties reported for C1P include, on the one hand, a positive effect on cell proliferation and cell survival [4,5] and, on the other hand, the control of cytosolic phospholipase A2 (cPLA2) activity [6-9]. Of note, however, neither knocking down the *Cerk *gene [3] nor using a CERK inhibitor [10] could recapitulate these findings, which suggests compensation by other C1P pools that do not depend on CERK for their synthesis.

The physiological role of CERK and its relevance to disease is only starting to be addressed. Using a gene knockdown strategy Igarashi and coworkers have shown preliminary evidence for a role of CERK in emotional behavior [11]. Based on ex- vivo work with CERK-deficient endothelial cells together with use of the CERK inhibitor NVP-231 [10] our laboratory has recently proposed a role for CERK in the regulation of angiogenesis [12]. CERK may also be relevant to immune cell biology. In fact, neutrophils represent one of the first cell types where CERK/C1P were described [13-15] and subsequently shown to promote phagolysosome formation [16]. More recently, the study of C1P/CERK in mast cells has suggested their function in the degranulation process [17]. Consistently, in a model of passive cutaneous anaphylaxis, *Cerk*^-/- ^animals were partially protected [3]. However, *Cerk*^-/- ^responded similarly to control littermates during a model of active cutaneous anaphylaxis [3]. In fact, work in vitro with CERK-deficient primary bone marrow derived mast cells or with the NVP-231 inhibitor failed to clarify a putative role of CERK/C1P in mast cell biology [3,10]. In an antigen-induced arthritis model, *Cerk*^-/- ^mice were not protected, thus inconsistent with the hypothesis that C1P made by CERK is key for cPLA2 activation in vivo -- parallel pharmacological inhibition of cPLA2 could indeed dampen disease development [3]. In sharp contrast, absence of CERK prevented *Cerk*^-/- ^animals from mounting an effective response against a pulmonary insult by Streptococcus pneumoniae [3]. Altogether, although CERK seems to play a role in inflammation, the overall picture remains contrasted and no consensus has emerged yet.

The study reported here was undertaken to examine T-cell specific responses in the absence of CERK. To this aim we made use of the established DO11.10 RAG1^-/- ^background [18-20]. In DO11.10 mice all thymocytes and peripheral T cells express the transgenic TCR receptor from a T cell hybridoma, DO11.10, that recognizes chicken ovalbumin (OVA) in the context of I-A^d ^class II MHC molecules. Furthermore, in DO11.10/RAG1^-/- ^mice, the endogenous TCR α-chains are eliminated, and T cells can only express the transgenic receptor. This model has proven useful for studying T-cell specific responses. Therefore, we backcrossed *Cerk*^-/- ^mice with DO11.10/RAG1^-/- ^mice to probe the effect of CERK ablation on T-cell activation. In parallel, we also backcrossed *Sphk1*^-/- ^and *Sphk2*^-/- ^to the same background for comparison.

## Methods

### Generation of DO11.10 RAG1^-/- ^mice strains

Mice deficient in either CERK, SPHK1 or SPHK2 were backcrossed with DO11.10 RAG1^-/- ^mice; all strains were on a Balb/C background. Deficiency for CERK, SPHK1 or SPHK2 was analyzed by RT-PCR as previously described [3,21]. For examination of the expression of the transgenic TCR receptor, blood was taken from the retro-bulbar complex, erythrocytes were lysed and T cells were stained with an anti KJ 126- FITC conjugated antibody (Caltag) and analyzed by flow cytometry (Becton Dickinson Excalibur).

### Ovalbumin challenge in vivo

On day 0 and 5, mice were sensitized by intraperitoneal injections of alum-precipitated ovalbumin (OVA) containing 100 μg OVA (Sigma) absorbed to 2 mg Al(OH)_3 _(Alu Gel S (2% Al(OH)_3_, Serva) and diluted with saline to a volume of 0.2 ml.

### Splenocyte isolation, culture and ovalbumin treatment

After immunization with OVA as described above, spleens were isolated and single splenocyte suspensions were prepared by lysing erythrocytes with FACS Lysing Solution (Becton Dickinson). One million cells/well of splenocytes were seeded in 96 well plates (Becton Dickinson), and incubated with 10 μg/ml OVA_323-339 _peptide for 3.5 hours in RPMI1640 supplemented with 100 U/ml penicillin, 100 μg/ml streptomycin sulfate and 10% FBS. After the incubation, culture supernatant were harvested, and kept at -20°C until cytokine measurement.

### Cytokine measurements

For parallel determination of cytokines in serum multiplex reagents from BioRad were used; analyses were conducted using a BioPlex 200 System, the BioPlex Pro reagent kit and human standards group I + II. Cytokine concentrations in the samples were calculated from calibration curves using the software XL-fit (IDBS Ltd.). IL-4 levels in culture medium of isolated splenocytes were measured by enzyme-linked immunosorbent assay (ELISA), following the manufacturer's instructions (R&D Systems).

### Asthma model

On days 0 and 5, mice were sensitized by intraperitoneal injection of alum-precipitated ovalbumin (cf above) and diluted with saline to a volume of 0.2 ml. On day 0 and 13, 200 μl blood was collected by puncture of the retro-bulbar plexus under isoflurane anesthesia. Sera were prepared and stored frozen at -20°C until analyzed for OVA-specific IgE and IgG1in serum. On day 12, the animals were challenged with aerosolized OVA (1.0% wt/vol in PBS) for 60 min, twice. The aerosol was generated by a "Small Particle Aerosol Generator" (SPAG-2, Serotherapeutisches Institut) driven by compressed air at 6 l/min. Under these conditions the median particle diameter was 0.5 μm. Forty eight hours after the first aerosol exposure, mice were euthanized with pentobarbital (Nembutal 70 μl/mouse i.p.). The trachea was trimmed free of connective tissue and a cannula was inserted. The lungs were lavaged with 3 × 1 ml saline at 37°C instilled through the tracheal cannula by a syringe. After five gentle massages of the thorax, approximately 3 × 0.7 ml brochoalveolar lavage fluid (BAL) was retrieved from the lungs and collected in an ice-cold plastic tube. The cells were counted using a Sysmex KX-21 cell counter (TOA). Differential cell counts were performed on cytocentrifuge preparations (Labofuge A; Heraeus) and stained with May-Grunwald/Giemsa. Differential cell counts were determined from at least 500 cells, and calculated per ml BAL fluid. Serum OVA-specific IgE and IgG1 levels were determined in mouse sera by ELISA. For the determination of OVA-specific IgE, 100 μl of rat anti mouse IgE heavy chain antibody (LO-ME-3, Serotec) at a concentration of 5 μg/ml bicarbonate buffer, pH 9.6, were added to each well of ELISA microtiter plates (cert. Maxisorp, Nunc). The plates were incubated at 4°C for 3 hrs then washed three times with wash buffer + 1% FCS. One hundred-μl of OVA reference serum (100 relative units/ml) or test sera diluted in wash buffer + 1% FCS in threefold steps were distributed into each well and incubated for 2 hrs at room temperature. Washing was repeated three times before 100 μl of biotinylated OVA (1:6000) in wash buffer + 1% FCS were distributed into each well and incubated over night at 4°C. After washing three times, 100 μl horseradish peroxidase avidin D (1:3000, Vector Labs) was added for 2 hrs at room temperature. Plates were washed three times with wash buffer followed three times with substrate buffer before adding 100 μl of the chromogen (5 mg orthophenyldiamine (Sigma)/10 ml phosphate citrate buffer containing 0.003% H_2_O_2_) into each well and incubating them for fifteen minutes at room temperature. The reaction was stopped with 50 μl 4N H_2_SO_4 _and the OD 492 nm read using a microtiter spectrophotometer (TECAN Spectra). The ODs of the serum samples were related to the standard curve and the amount of OVA-specific IgE were calculated. For determination of OVA-specific IgG1, 100 μl of OVA at a concentration of 2 μg/ml bicarbonate buffer, pH 9.6, were added to each well of ELISA microtiter plates (cert. Maxisorp, Nunc). The plates were incubated at 37°C for 2 hrs and stored until use at 4°C. Then the plates were washed three times with wash buffer. One hundred-μl of OVA reference standard serum or test sera diluted in PBS + 1% FCS in threefold steps were distributed into each well and incubated for 2 hrs at 37°C. Washing was repeated three times before 100 μl of biotinylated anti-mouse IgG1 (The Binding Site, diluted 1:10,000) were added to each well and incubated for 60 min at 37°C. After repeated washings, 100 μl of horseradish peroxidase (Vector Lab.) was added for 60 min at 37°C, and then washed three times with wash buffer. The assay was developed using the same procedure as for OVA-specific IgE measurement. PGE_2 _in BALF was measured by ELISA (R&D Systems) according to the manufacturer's instructions.

## Results

### Reduced IL-4 levels in *Cerk*^-/- ^DO11.10/RAG 1^-/- ^mice challenged with ovalbumin

T cell activation triggers signaling mechanisms that induce the production and secretion of cytokines. Functionally, cytokines have been classified as being either pro-inflammatory (T_H_1 type) or anti-inflammatory (T_H_2 type) depending on the final balance of their effects on the immune system. For analysis, we selected a panel of cytokines belonging to these two categories, namely IL-2, TNF-α, INF-γ (T_H_1) and IL-4, IL-13 and IL-5 (T_H_2).

**Figure 1:**
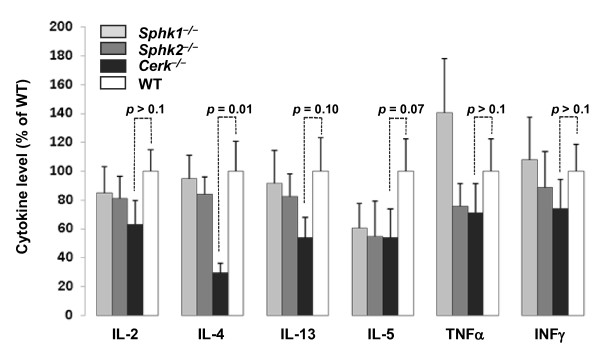
**Cytokine release upon OVA challenge in DO11.10 RAG1^-/- ^mouse lines**. *Cerk*^-/-^, *Sphk1*^-/- ^and *Sphk2*^-/- ^and WT mice all in the genetic background *DO11.10 RAG1*^-/-^, were challenged with OVA as described in the Experimental section. Blood samples were collected after 2 h and 4 h for cytokine measurement and serum was prepared. Serum cytokine levels shown are the mean ± SD of the 2 h together with the 4 h measurements expressed as a percentage of WT levels, from 3 independent experiments with a total of 24 animals per group.

The four strains (wild-type (WT), *Cerk*^-/-^, *Sphk1*^-/- ^and *Sphk2*^-/-^), all in the DO11.10 RAG1^-/- ^background, were challenged with ovalbumin. Blood samples were collected at 2 h and 4 h post challenge and the levels of the above-mentioned cytokines in the serum was measured using multiplex analysis (Fig. 1). IL-4 levels in the serum were reduced by 70% in *Cerk*^-/- ^compared to WT (p = 0.01) and compared to both SPHK-deficient animals. There was also a clear trend towards reduction of secreted IL-13 in *Cerk*^-/- ^animals (- 45%; p = 0.10 *vs *WT), which was observed neither with *Sphk1*^-/- ^nor with *Sphk2*^-/- ^animals. A trend for reduction of IL-5 levels (-45%; p = 0.07 in *Cerk*^-/- ^*vs *WT) was seen for all 3 knock-out strains. Levels for all other measured cytokines were not significantly different from WT levels.

**Figure 2:**
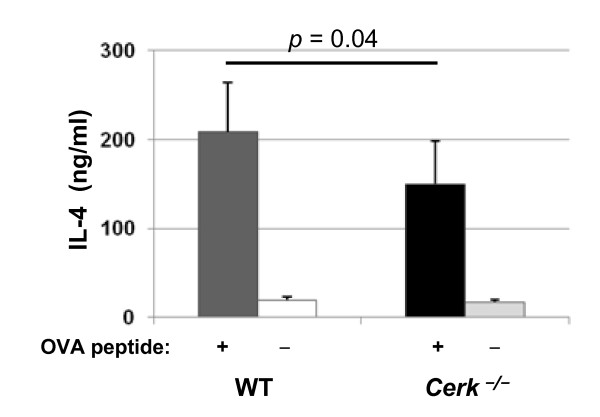
**IL-4 release in OVA challenged isolated splenocytes**. Splenocytes from *DO11.10 RAG1*^-/- ^and from *Cerk*^-/- ^*DO11.10 RAG1*^-/- ^mice were isolated following ovalbumin challenge *in vivo *as described in the Experimental section. Cultured splenocytes were then incubated with 10 μg/ml OVA_323-339 _peptide or with saline (control) for 3.5 hours and IL-4 levels were measured in cell culture supernatant. The data shown represent the mean ± SD of measurements obtained from 8 animals (OVA-treated) and 2 animals (controls). In this experiment it was not possible to match the age of the animal population optimally: *DO11.10 RAG1*^-/- ^were 8 weeks of age at start of the experiment whereas *Cerk*^-/- ^*DO11.10 RAG1*^-/- ^mice were between 11 and 14 weeks of age.

To confirm the data obtained in vivo, we measured IL-4 secretion ex- vivo, from CERK-deficient DO11.10/RAG 1^-/- ^splenocytes. After 3.5 h of ovalbumin peptide challenge in culture, the levels of IL-4 secreted by *Cerk*^-/- ^cells was reduced by 30% (p = 0.04) compared to WT cells (Fig. 2).

### *Cerk*^-/- ^mice are not protected in an ovalbumin-induced asthma model

**Figure 3:**
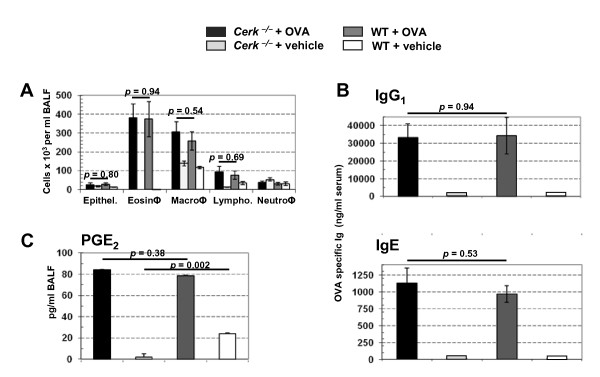
**Asthma model**. *Cerk*^-/- ^and WT mice were exposed to OVA-aerosol as described in the Experimental section. BAL fluid and blood were collected at 48 hours after OVA challenge. The number of infiltrated inflammatory cells (A), OVA-specific IgG1 and IgE in serum (B) and PGE_2 _in BAL fluid (C) are represented as the mean ± SEM of 8 animals per group.

IL-4, IL-5 and IL-13 are key cytokines for adaptive immunity, produced by CD4^+ ^T cells of the T_H_2 type. The overall reduced production of these cytokines in *Cerk*^-/- ^DO11.10/RAG1^-/- ^mice therefore suggested a possible contribution of CERK to T_H_2 cell biology. In addition, because IL-4 and IL-13 are important regulators of immediate hypersensitivity reactions by inducing B cell immunoglobulin (Ig) heavy chain class switching to the IgE isotype, the strong depletion of IL-4 levels in absence of CERK might have broader implications. Therefore, we submitted *Cerk*^-/- ^mice to an ovalbumin-induced asthma model, where cytokines such as IL-4 and IL-13 are playing key roles. We measured leukocyte infiltration, immunoglobulin levels in the serum as well as inflammatory mediator release. When assessed 2 days after the final ovalbumin aerosol challenge, the numbers of eosinophils, macrophages, lymphocytes and neutrophils, harvested from the bronchoalveolar lavage fluid, were indistinguishable in WT and *Cerk*^-/- ^animals (Fig. 3A). Similar observations were made for IgG1 and IgE levels (Fig. 3B) and for prostaglandin (PG) E_2 _levels (Fig. 3C).

## Discussion

**Figure 4:**
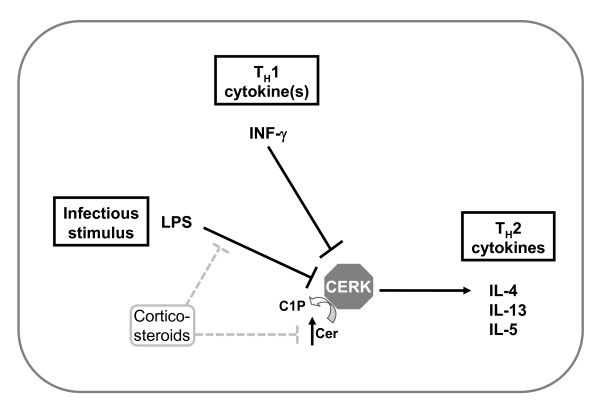
**CERK and T_H_1/T_H_2 cytokines**. This schematic representation summarizes the current knowledge of the interplay between CERK and cytokines. The T_H_1 cytokine INF-γ as well as LPS can repress CERK at transcriptional level in primary mouse macrophages which indicates that a T_H_1 response may require initial down-regulation of CERK; this can be reversed by corticosteroids. The present work provides evidence that T_H_2 cytokine production, IL-4 in particular, are dependent on CERK. Altogether, this suggests that CERK may polarize immune responses towards the T_H_2 type.

The results obtained with *Cerk*^-/- ^compared to *Sphk1*^-/- ^and *Sphk2*^-/-^, in the DO11.10/RAG1^-/- ^genetic background, revealed that IL-4 production and/or release, after T-cell activation, is dependent on CERK (Fig.1 and Fig. 2). Furthermore, even if this did not reach statistical significance, the same experiments also revealed that IL-13 and IL-5 levels are reduced in absence of CERK (Fig. 1). Altogether, this suggested that CERK may play a role in T_H_2 cytokine production. Remarkably however, *Cerk*^-/- ^animals (in the normal Balb/C background) were not protected in an asthma model where T_H_2 cells are considered to be a driving force (Fig. 3). It is possible that reduction of IL-4 levels in absence of CERK has not been sufficient to limit the immune response; equally possible might be the mechanistic redundancy between cytokines (e.g. IL-4 and IL-13; levels of the latter are only partially reduced in absence of CERK; Fig. 1). An alternative explanation might be differential compensation in a background of C1P reduction (due to absence of CERK) in the two models. Readouts in DO11.10 were taken after 2 to 4 h whereas analysis in the asthma model took place after 48 h; compensatory C1P synthesis (by unknown mechanisms, cf ref. [2]) might be limiting only for short period of times. Altogether, despite a clear cut influence on IL-4 levels, to what extent CERK may participate in T_H_2 cell biology remains elusive. Interestingly, however, infectious reagents such as LPS or the T_H_1 cytokine INF-γ can inhibit CERK at mRNA levels in primary macrophages (P. Rovina and F. Bornancin, unpublished data) thus supporting the notion that CERK activity may be positively linked to the T_H_2 rather than to the T_H_1 phenotype (Fig. 4).

Basal levels of PGE_2 _in BAL fluid were significantly reduced in control *Cerk*^-/- ^compared to control WT mice (Fig. 3C). In fact, this observation provides first in vivo support to the hypothesis that CERK signals to cPLA2, which has already been well validated in vitro [6-9]. It also indicates that the contribution of CERK/C1P to cPLA2 function might be best evidenced when cPLA2 is sub-optimally activated. Other mechanisms may prevail under developing acute or chronic inflammation e.g. during the asthma model presented here.

In addition to the asthma model of the present work, other disease models of the lung were tested with *Cerk*^-/- ^mice. A smoke-induced chronic obstruction model, where increased sensitivity in the absence of CERK may be expected because of up-regulated ceramide levels [3], failed to reveal significant differences between *Cerk*^-/- ^and WT control littermates (data not shown). A bleomycin-induced fibrosis model, where IL-13 is key, also did not show differences between *Cerk*^-/- ^and WT control littermates (data not shown). In fact, the sole lung pathology where we observed a statistically different behavior in *Cerk*^-/- ^compared with WT is an infection model with S. pneumoniae, as already reported [3]. During this fulminant infection, absence of CERK led to drastic worsening of the disease resulting in early mortality. We initially speculated on increased ceramide levels in absence of CERK leading to premature neutrophil apoptosis and inefficient first line defense [3]. Should the T cells be polarized by CERK towards the T_H_2 type (Fig. 4), the opposite hypothesis could also be considered, i.e. hyperactivation of host defenses in *Cerk*^-/- ^mice might have led to poor control of the infection. In fact, the dynamic (as opposed to "established" i.e. in KO animals) down-regulation of CERK might represent a negative feedback mechanism. Clearly, further experiments are required to understand the contribution of CERK to inflammation. In light of available data including the results of the present study, CERK may play a 'fine tuning' role in inflammatory processes.

## Abbreviations

BAL: (bronchoalveolar); CERK: (ceramide kinase); C1P: (ceramide-1-phosphate); ELISA: (enzyme linked immunosorbent assay); Ig: (immunoglobulin); IL: (interleukin); INF: (interferon); OVA: (ovalbumin); SPHK: (sphingosine kinase); (T_H_): T Helper; WT: (wild-type).

## Competing interests

All authors are present (SN, TB, AB, FB) or former (NU) employees of Novartis

## Authors' contributions

SN, NU, TB, AB and FB conceived and designed the experiments; SN and NU performed the experiments, SN, AB and FB performed the statistical analyses; FB wrote the manuscript. All authors read and approved the final manuscript.
